# Comparison of topical and infiltration anesthesia for orthodontic mini-implant placement

**DOI:** 10.1590/2176-9451.19.2.076-083.oar

**Published:** 2014

**Authors:** Matheus Miotello Valieri, Karina Maria Salvatore de Freitas, Fabricio Pinelli Valarelli, Rodrigo Hermont Cançado

**Affiliations:** 1 MSc in Orthodontics, Ingá College (UNINGÁ).; 2 Coordinator of the Master's program in Orthodontics, Ingá University (UNINGÁ).; 3 Adjunct professor, Department of Orthodontics, Master's program, Ingá College (UNINGÁ).

**Keywords:** Anesthetics, Orthodontics, Dental implants, Orthodontic anchorage procedures

## Abstract

**Objective:**

To compare the acceptability and effectiveness of topical and infiltration anesthesia for placement of mini-implants used as temporary anchorage devices.

**Methods:**

The sample comprised 40 patients, 17 males and 23 females, whose mean age was 26 years old and who were all undergoing orthodontic treatment and in need for anchorage reinforcement. Mini-implants were bilaterally placed in the maxilla of all individuals, with infiltration anesthesia on one side and topical anesthesia on the other. These 40 patients completed two questionnaires, one before and another after mini-implant placement and pain was measured through a visual analog scale (VAS). The data collected were analyzed using descriptive statistics and the measurements of pain were compared by means of the non-parametric test of Mann-Whitney.

**Results:**

It was found that 60% of patients felt more comfortable with the use of topical anesthesia for mini-implant placement; 72.5% of patients described the occurrence of pressure during placement of the anchorage device as the most unpleasant sensation of the entire process; 62.5% of patients felt more pain with the use of topical anesthesia.

**Conclusion:**

It was concluded that patients had less pain with the use of infiltration anesthesia, and also preferred this type of anesthetic.

## Abstract (pt)

**Objetivo:**

comparar a aceitabilidade e a efetividade do uso de anestésico tópico e anestésico infiltrativo para inserção de mini-implantes ortodônticos, utilizados como dispositivos de ancoragem temporária.

**Métodos:**

foram selecionados 40 pacientes, sendo 17 do sexo masculino e 23 do sexo feminino, com idade média de 26 anos, todos em tratamento ortodôntico e necessitando de reforço de ancoragem. Em todos os indivíduos foram instalados mini-implantes bilateralmente em maxila, sendo em um dos lados com anestesia infiltrativa e do lado oposto com anestesia tópica. Esses 40 pacientes responderam dois questionários, sendo um pré- e outro pós-operatório, e foram obtidos índices de dor por meio da escala visual análoga (VAS). Os dados coletados foram analisados por meio de estatística descritiva e os índices de dor foram comparados por meio do teste não-paramétrico de Mann-Whitney.

**Resultados:**

verificou-se que 60% dos pacientes se sentiram mais confortáveis com a utilização de anestesia tópica para a inserção dos mini-implantes; 72,5% dos pacientes apontaram a pressão durante a inserção do dispositivo de ancoragem como a sensação mais desagradável de todo o processo; 62,5% dos pacientes sentiram mais dor com o uso de anestesia tópica.

**Conclusão:**

concluiu-se que os pacientes apresentaram menor índice de dor com o uso de anestesia infiltrativa e que, também, preferiram esse tipo de anestésico.

## INTRODUCTION

According to Newton's third law, every action has a reaction of equal magnitude and towards its opposite direction. Therefore, when a force is applied with the purpose of achieving orthodontic movement, the teeth used as support (anchorage) will have a reaction with the same intensity towards the opposite direction, which, in most cases, may generate undesirable effects.

In order to avoid such undesirable effects in orthodontic mechanics, the clinician should carefully plan the anchorage to be employed during treatment. However, some types of anchorage directly depend on patient's compliance, which may compromise the final results.

With a view to solving the issues related to anchorage, dentists have had the possibility of using devices that enable skeletal support for tooth movement.

Mini-implants and mini-plates are among the skeletal anchorage devices most commonly used for orthodontic mechanics. The use of mini-plates and mini-implants enable dental movement to be safely performed, many times, without undesirable side effects, at the vertical, transverse, and anterior-posterior planes.^1^

The orthodontic loads of continue and unidirectional nature and of low magnitude are not capable of generating osteolytic activity on the bone interface of the implant.^2,3^

Assessment of patients' acceptance factors regarding the use of mini-implants during orthodontic treatment reveals that the need for infiltrative anesthesia is one of the factors that patients reject the most.^4^ Additionally, the association with osseointegrated implants is another factor that contributes to increase the rejection and fear of patients with regard to the use of mini-implants. Several topical anesthetics are available to be used before minor dental procedures are performed and they are largely accepted by the patients.

The ideal topical anesthetic would promote complete anesthesia, with fast action onset and without any side effects. The agents currently available, however, are only close to this ideal.^5^

The possibility of placing mini-implants with the use of topical anesthetic only, has already been suggested in the literature.^4,6,7^ Some authors have reported that mini-implants could be successfully and comfortably placed with the use of topical anesthetic, only.^8^ Two types of topical anesthetics used for mini-implant placement have been compared, and one of them showed highly satisfactory results.^9^ Additionally, it has been proved that 90% of patients undergoing mini-implant placement with the aid of topical anesthesia only, would accept to have mini-implants replaced , if necessary. In this study, 40% of patients reported not having felt any type of pain during placement of the mini-implant, while 20% reported mild pain.^10,11^

However, no study has been conducted to compare the acceptability and effectiveness of infiltrative and topical anesthetics. Thus, the aim of this study was to compare, by means of pre and post-operative questionnaires answered by the patients, the acceptability and discomfort of infiltrative and topical anesthetics used for placement of mini-implants as skeletal anchorage in Orthodontics.

## MATERIAL AND METHODS

### Sample

This study was approved by the Ingá School of Dentistry Institutional Review Board. Sample calculation was based on alpha error of 5% and beta error of 20%, so as to reach a power test of 80% in order to detect a significant difference of 1.00 cm in VAS scale, with a standard deviation of 1.5, resulting in 36 subjects required for each group.

The study sample comprised 40 patients, 17 males and 23 females, with mean age of 26 years old (not younger than 14, not older than 45 years old). All patients underwent orthodontic treatment and needed bilateral absolute anchorage through mini-implants in the maxilla.

This was a prospective study of which patients were treated in the Orthodontic Clinics of the Masters Course of the Ingá School of Dentistry, and required the placement of bilateral maxillary mini-implants while the study was being carried out, until the number of 40 subjects was obtained.

All patients had the mini-implants placed at the same appointment. The anesthetic was used alternately, that is, the topical anesthetic was applied on one side, while the infiltrative anesthetic was used on the other side. The anesthesia was applied by one examiner who had been previously trained by a Professor of the Masters Course in Orthodontics who, in turn, has extensive expertise in the mini-implant placement either with infiltrative or topical anesthesia.

Two questionnaires (one before and one after mini-implant placement) were given to each patient in order to compare the efficiency of each anesthetic.

### Anesthetics

Patients underwent two different anesthetic procedures for mini-implant placement:

» Infiltrative - lidocaine hydrochloride+ epinephrine 1:100,000 (Alphacine 100, DFL Commerce and Industry, Jacarepaguá-RJ, Brazil) applied where the mini-implant was placed, with the aid of a 0.30 x 21 mm gingival needle (Terumo) in the mucosa area, only, with 1/5 of the tube being injected. Mini-implant placement was performed 2 minutes after the infiltrative anesthesia was applied.
» Topical: on the opposite side, topical anesthetic gel with 20% lidocaine ( Relva Dermatological Pharmacy, Campo Grande-MS, Brazil) was applied for 7 minutes on the area of the mucosa that received the mini-implant. If the patient reported great pain during mini-implant placement with topical anesthetic, the procedure would be interrupted and the infiltrative anesthetic would be used.

### Mini-implants

Self-drilling mini-implants 6 mm in length and 1.5 mm in diameter (Conexão, São Paulo, Brazil) were used in this study. To place the implants, a surgical kit with hand key (Conexão, São Paulo, Brazil) was used.

### Mini-implant placement

All patients included in this study were submitted to the following protocol:

» The patient answered the pre-operative questionnaire.
» Drying with air jet and relative isolation was performed with cotton rolls to move the lip away from the area where the mini-implant would be placed under topical anesthetic.
» Topical anesthesia with 20% lidocaine gel was applied on a cotton pellet placed onto the mucosa where the mini-implant would be placed. The gel had to be kept on the mucosa for 7 minutes.
» Removal of excess gel with the aid of a gauze.
» Mini-implant placement.
» On the opposite side, infiltrative anesthesia with Alphacaine 100 (lidocaine hydrochloride+ epinephrine1:100,000) was applied in the area where the mini-implant would be placed.
» Mini-implant placement 2 minutes after anesthesia.
» The patient answered the post-operative questionnaire.

The type of anesthesia that was applied first should be alternated for every other patient. All mini-implants were placed without the need for previous perforation.

### Questionnaires

The patients included in the sample were submitted to questionnaires comprised of objective questions before and after mini-implant placement. (Questionnaires are available at http://dpjo.dentalpresspub.com/editions/v19n2/076-083/).

The visual analogue scale (VAS),^12^ which is largely used for pain quantification, was employed in question number 4 of the post-operative questionnaire.

### Statistical analysis

A descriptive statistical analysis was performed. The comparison of the VAS results for topical and infiltrative anesthetics was performed by means of the Mann-Whitney non-parametric test.

To evaluate the sexual dimorphism of the responses of VAS, the Mann-Whitney non-parametric test was applied.

All tests were performed with the aid of Statistica software (Statistica for Windows, version 7.0, Statsoft, 2005). The level of significance was set at P < 0.05.

## RESULTS

### Pre-operative results

**Figure 1:**
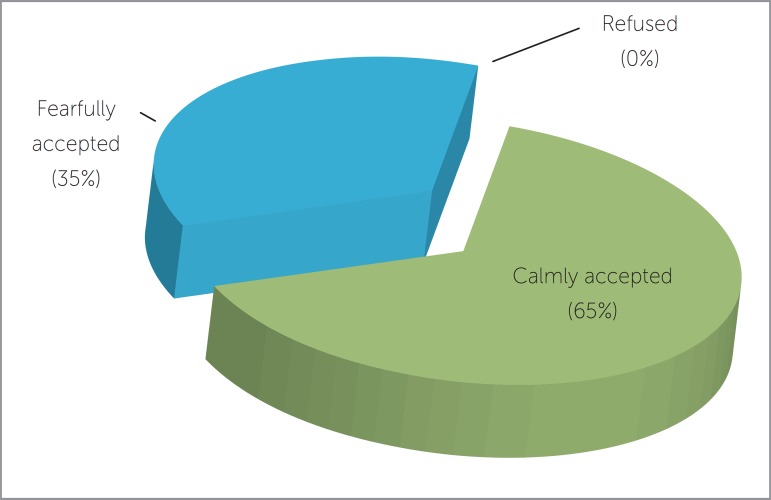
Answers to question number one of the pre-operative questionnaire: "When your orthodontist proposed mini-implant installation, how did you react?"

**Figure 2:**
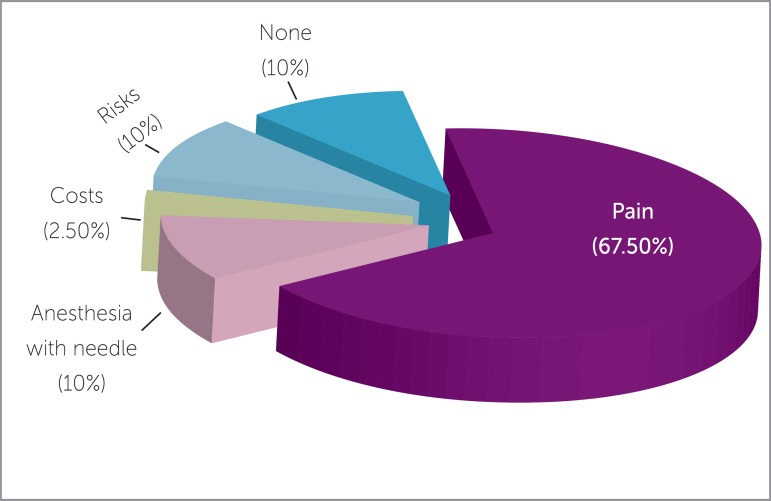
Answers to question number two of the pre-operative questionnaire: "After the dentist proposed mini-implant installation, which was your main doubt regarding the procedure?"

Out of the 40 patients comprising the sample, 65% answered that they calmly accepted the proposal for mini-implant placement (Fig 1). 67.5% of patients reported that their main concern about the procedure was with regards to pain (Fig 2).

**Figure 3:**
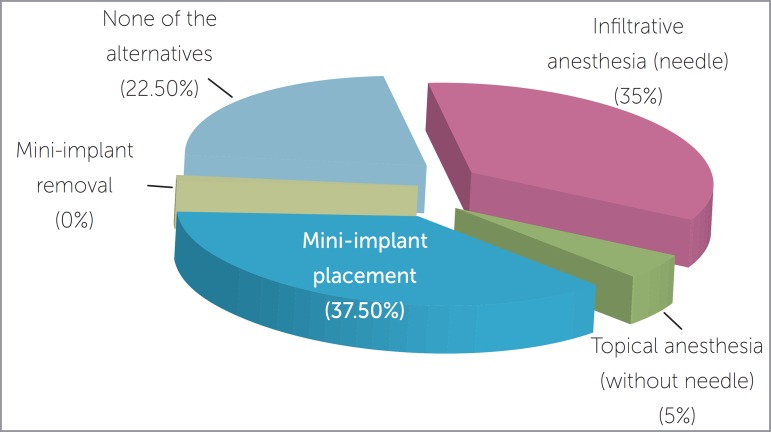
Answers to question number three of the pre-operative questionnaire: "Which of these procedures make you more fearful about installing the mini-implant?"

When asked about the most worrying procedure, the responses "Mini-implant placement" and "Infiltrative anesthesia (needle)" were the most frequent ones with 37.5% and 35% respectively (Fig 3).

**Figure 4:**
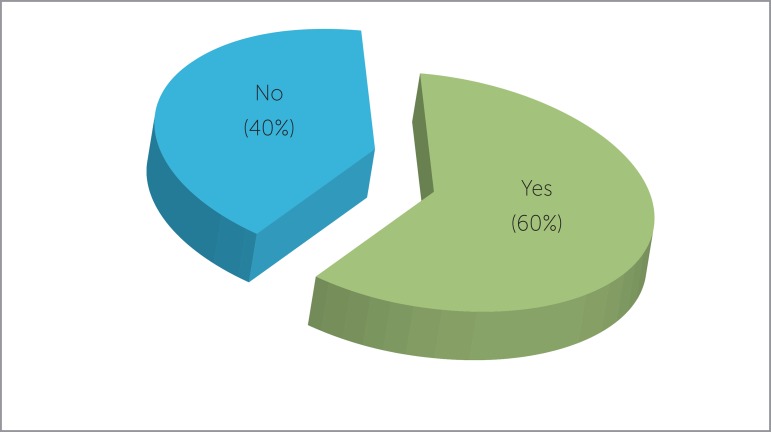
Answers to question number four of the pre-operative questionnaire: "Does the fact of using the topical anesthesia (without needle) make you more comfortable regarding the mini-implant installation?"

Sixty percent (60%) of patients claimed to feel more comfortable towards having mini-implants placed with topical anesthesia (Fig 4).

### Post-operative results

**Figure 5:**
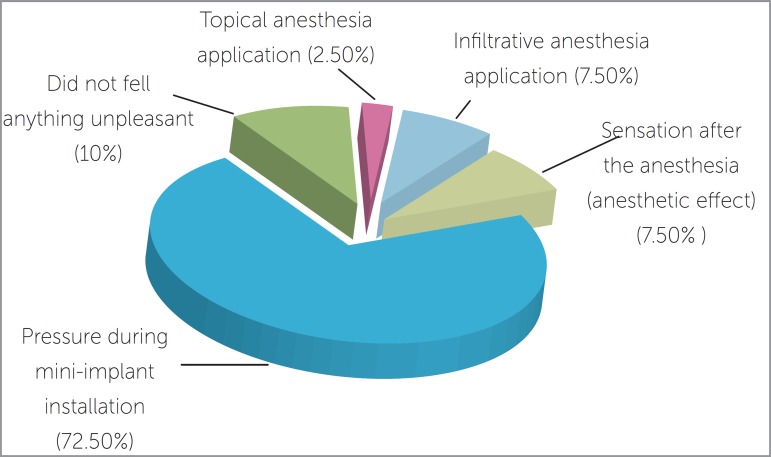
Answers to question number one of the post-operative questionnaire:"Which was the most unpleasant sensation related to mini-implant installation?"

**Figure 6:**
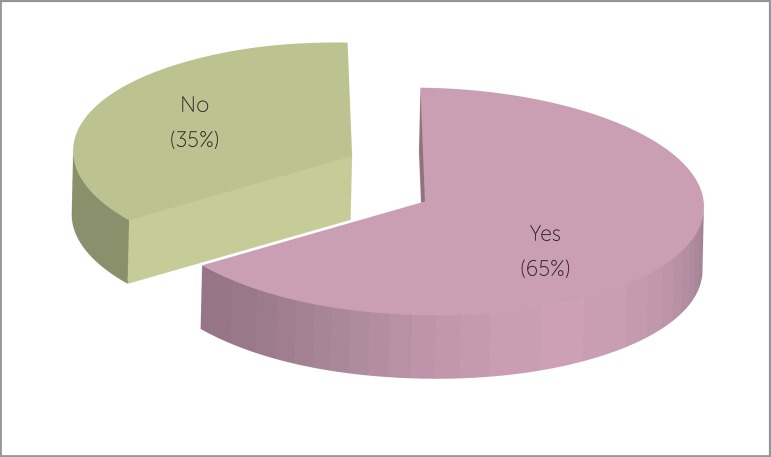
Answers to question number two of the post-operative questionnaire: "Did you feel pain at any moment of the mini-implant installation?"

**Figure 7:**
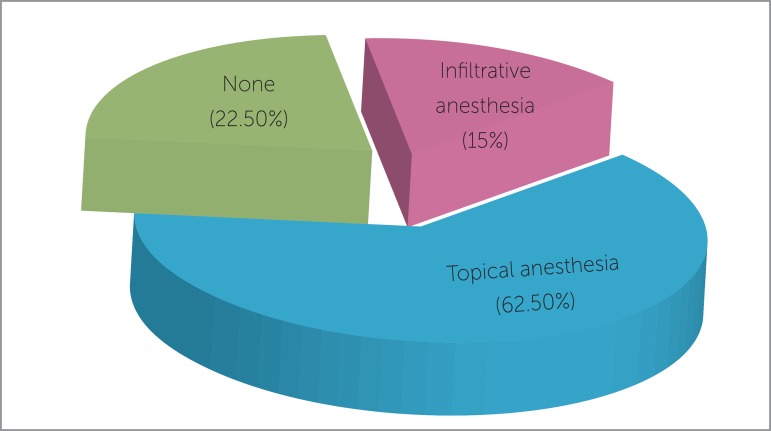
Answers to question number three of the post-operative questionnaire: "With which type of anesthesia did you feel more painful sensation?"

Twenty-nine patients (72.5%) reported that pressure during mini-implant placement was the most unpleasant sensation they felt during treatment (Fig 5). When asked whether they felt pain at any moment during mini-implant placement, 65% of patients answered affirmatively, while 35% claimed that they did not feel any pain (Fig 6). As for the type of anesthesia that caused the most severe pain, 62.5% of patients answered that pain was worse under topical anesthesia ( Fig 7).

**Figure 8:**
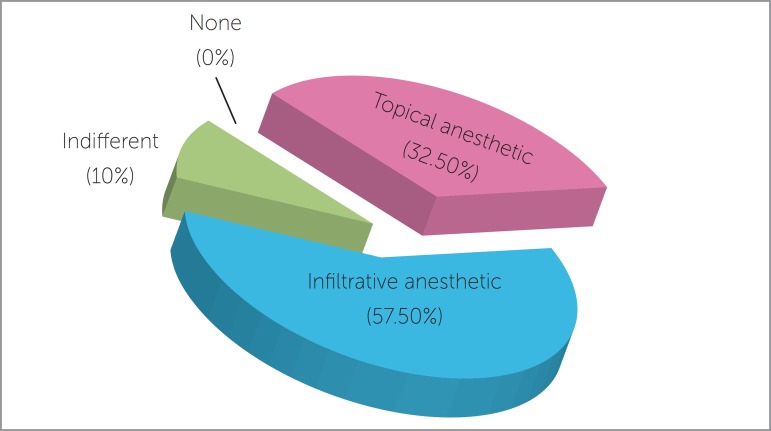
Answers to question number five of the post-operative questionnaire: "By comparing topical and infiltrative anesthesia, which type did you prefer?"

According to the responses obtained, the anesthetic of choice of the majority of patients was the infiltrative anesthetic (23 patients), while 13 patients preferred the topical anesthetic and 4 patients reported they did not have any preference regarding the anesthetic used (Fig 8).

**Figure 9:**
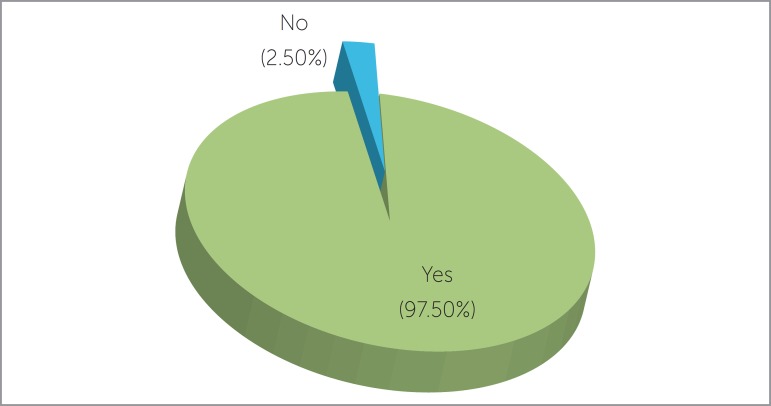
Answers to question number six of the post-operative questionnaire: "If necessary, would you be submitted to mini-implant installation again?"

Only one patient claimed to refuse having mini-implant replaced, if necessary (Fig 9).

Mean values of pain were obtained by assessment of the visual analogue scale used in question 4 of the post-operative questionnaire. The infiltrative anesthetic obtained a mean value of 0.3125, while the topical anesthetic obtained a mean value of 3.0875. These data were compared through the Mann-Whitney non-parametric test, with statistically significant differences. As a result, mini-implants placed with topical anesthetic caused significantly more pain than those placed with topical anesthesia, as shown by the VAS scale (Table 1). When the sample was divided according to sex, the mean values obtained were 0.2647 with the infiltrative anesthesia and 3.6764 with the topical anesthesia in males; whereas females had mean values of 0.3478 with the infiltrative anesthesia and 2.6521 with the topical anesthesia. These data were also submitted to the Mann-Whitney non-parametric test, without statistically significant differences (Table 2).

**Table 1:** Comparison of VAS results through the Mann-Whitney non-parametric test.

Variable	Infiltrative anesthesia (n = 40)	Topical anesthesia (n = 40)	p
	Mean ± SD	Mean ± SD	
VAS	0.31 ± 0.64	3.08 ± 2.54	0.000*

* Statistically significant for P < 0.05.

**Table 2:** Sexual dimorphism assessment by comparison of VAS results through the Mann-Whitney non-parametric test.

Variable	Male (n = 17)	Female (n = 23)	p
	Mean ± SD	Mean ± SD	
Infiltrative	0.26 ± 0.66	0.34 ± 0.64	0.671
Topical	3.67 ± 2.29	2.65 ± 2.67	0.112

## DISCUSSION

### Discussion of the method

Many authors have suggested the possibility of using topical anesthetic for mini-implant placement with a view to obtaining the analgesia required for complete insertion of the anchorage device without blocking the sensibility of the surrounding structures, thus, reducing the chances of damages if the mini-implants reaches these structures.^4,6,11^ In the present study, the use of topical gel anesthetic (20% lidocaine)^10,11^ was chosen because it reaches good levels of analgesia, can be easily handled and does not cause tissue damage, as previously reported by the literature. The application protocol used in this study also followed the recommendations of a previous study,^10^ that is, the gel was kept in contact with the mucosa for 7 minutes under relative isolation and care so that it did not surpass the area of interest.

With regard to the infiltrative anesthetic, lidocaine hydrochloride + epinephrine 1:100,000 (Alphacaine 100^®^) was used due to the fact that it is largely employed in Dentistry with low toxicity rates and enough anesthetic effect. The amount of anesthetic used was of 1/5 of the tube, injected in the area of the mucosa where the mini-implant would be placed so as to allow a satisfactory anesthesia and prevent the surrounding structures from being anesthetized, as suggested by the literature.^13,14^

Self-drilling mini-implants (Conexão^®^), 6 mm in length and 1.5 in diameter were used with the aid of a surgical kit (Conexão^®^). The sequence of insertion was carried out in turns: half of times infiltrative anesthesia was the first procedure, while half of times topical anesthesia was applied first. The sides of insertion were also alternated in order to avoid any potential influences over patients' responses.

The use of topical anesthetic is very common before infiltrative anesthesia so as to decrease the discomfort in the application of the latter. However, such procedure was not carried out in this study. Pain was assessed while the mini-implant was being placed and not during anesthesia application, whether topical or infiltrative.

The mini-implants were bilaterally placed in the area between pre-molars and molars, on both sides of the same patient, so that the insertion area would not influence patient's pain sensitivity. The alternate order of use of the anesthetics for mini-implant placement also enabled the differences in sensitivity of both types of anesthetics to follow a single pattern of influence over the results.

Other factor that could have influenced the results was the anatomical differences of each patient, as they could alter pain threshold. Notwithstanding, because mini-implants were placed with both anesthetics in the same patient, this factor was practically annulled.

The visual analogue scale was used to record pain rates. It was chosen due to its easy clinical applicability and great power of pain measurement.^12^

### Discussion of results

In the present study, the acceptability of mini-implant placement was of 100% for all cases. However, 35% of patients answered they accepted with fear, while in another study 90% of patients answered "I immediately accepted because I totally trust my orthodontist". Nevertheless, this latter study comprised a considerably smaller sample (10 patients) and did not aim at evaluating different types of anesthesia, which could have influenced the data obtained.^4^

Infiltrative anesthesia was reported by 14 subjects as one of the most fearful procedures, followed by the fear of mini-implant placement, chosen by 15 patients. Sixty percent of patients reported that the use of topical anesthetic made them feel more comfortable with regard to the procedure, which proves that infiltrative anesthesia applied with the aid of a needle causes certain discomfort in a considerably number of patients,^15^ leading some of them to refuse being submitted to procedures of anchorage device placement.

After mini-implant placement, patients reported that the most unpleasant sensation they felt during the entire procedure was the pressure during placement, which was also observed by another study,^4^ but disagrees with what was found by Santos et al^11^ who reported that patients did not feel anything unpleasant.

Twenty-five patients pointed out that topical anesthesia caused the most painful sensation, proving that infiltrative anesthesia resulted in greater anesthetic effect for the patients of the sample.

Mini-implant placement could not be completed in three patients who received topical anesthetic. These patients reported severe pain, and for this reason, the procedure was discontinued, following the methods of this study. Later on, these same patients underwent infiltrative anesthesia and in two of them, the mini-implants were placed under infiltrative anesthesia. The other case was initiated by topical anesthetic. In the study conducted by Reznik et al,^9^ who compared two types of topical anesthetics, the failure rate (impossibility of finishing the installation under topical anesthesia) was of 71% (12 cases) when 20% benzocaine was used, whereas there was no failure when 20% lidocaine + 4% tetracaine + 2% phenylephrine anesthetic was used.

When patients were asked whether they would accept to have mini-implants replaced, 97.5% (39 patients) gave affirmative answers, while in the study conducted by Santos et al,^11^ 10% of patients would not accept it, and according to Brandão and Mucha,^4^ 10% of patients would not recommend this procedure to other patients.

The analysis of the data obtained with the visual analogue scale demonstrated that the mean values exhibited by the infiltrative anesthesia were minimum and significantly lower than those of the topical anesthesia (Table 1). Moreover, it could be observed that the discrepancy of pain values between both types of anesthetic was statistically significant. However, 42.5% of patients did not choose the infiltrative anesthesia as their procedure of choice (Fig 8), which demonstrates the rejection of most patients in regard to anesthetic procedures performed with the aid of needles.

The results of the visual analogue scale divided by sex demonstrated lower mean values for females when topical anesthetic was used. However, when the Mann-Whitney non-parametric test was applied to verify sexual dimorphism, the values did not prove to be statistically significant (Table 2).

### Clinical considerations

Failure was reported in three cases of mini-implant placement under topical anesthesia. Following the methods established for this study, infiltrative anesthesia was then applied and the anchorage device was installed. However, it was clear that, in all three cases of failure, the patients exhibited great anxiety while the procedure was being carried out, and after infiltrative anesthesia, they did not report any pain. The present study demonstrates that patients reported greater sensitivity in cases of mini-implant placement under topical anesthesia, without, however, reporting any discomfort during the anesthetic application. On the other hand, in cases of mini-implant placement carried out under infiltrative anesthesia, patients reported certain degree of discomfort during anesthetic application, but significant comfort during mini-implant placement, which must be assessed by both the dentist and the patient in the decision for which type of anesthetic should be employed.

Mini-implant placement under topical anesthesia seems to be a viable option in cases in which patients refuse to undergo infiltrative anesthesia in fear of the needle, especially in less anxious patients.

## CONCLUSION

Based on the results of the present study, it is reasonable to conclude that:

» The use of topical anesthetic results in more comfort to patients undergoing mini-implant placement procedures;
» Patients considered pressure during mini-implant placement as the most unpleasant sensation;
» Pain sensitivity of mini-implant placement with topical anesthetic was significantly greater than that of infiltrative anesthesia;
» Most patients submitted to mini-implant placement preferred the procedure under infiltrative anesthesia.
